# Global Relative Importance of Denitrification and Anammox in Microbial Nitrogen Loss Across Terrestrial and Aquatic Ecosystems

**DOI:** 10.1002/advs.202406857

**Published:** 2024-12-31

**Authors:** Gang He, Danli Deng, Manuel Delgado‐Baquerizo, Wenzhi Liu, Quanfa Zhang

**Affiliations:** ^1^ Hubei Key Laboratory of Wetland Evolution & Ecological Restoration, Wuhan Botanical Garden Chinese Academy of Sciences Wuhan 430074 P.R. China; ^2^ Hubei Field Observation and Scientific Research Stations for Water Ecosystem in Three Gorges Reservoir China Three Gorges University Yichang 443002 P.R. China; ^3^ Laboratorio de Biodiversidad y Funcionamiento Ecosistémico. Instituto de Recursos Naturales y Agrobiología de Sevilla (IRNAS) CSIC Av. Reina Mercedes 10 Sevilla E‐41012 Spain; ^4^ Danjiangkou Wetland Ecosystem Field Scientific Observation and Research Station Chinese Academy of Sciences & Hubei Province Wuhan 430074 P.R. China

**Keywords:** anammox, denitrification, eutrophication, global prediction, nitrogen removal

## Abstract

Denitrification and anaerobic ammonium oxidation (anammox) are the major microbial processes responsible for global nitrogen (N) loss. Yet, the relative contributions of denitrification and anammox to N loss across contrasting terrestrial and aquatic ecosystems worldwide remain unclear, hampering capacities to predict the human alterations in the global N cycle. Here, a global synthesis including 3240 observations from 199 published isotope pairing studies is conducted and finds that denitrification governs microbial N loss globally (79.8±0.4%). Significantly, anammox is more important in aquatic than terrestrial ecosystems worldwide and can contribute up to 43.2% of N loss in global seawater. Global maps for N loss associated with denitrification and anammox are further generated and show that the contribution of anammox to N loss decreases with latitude for soils and sediments but generally increases with substrate depth. This work highlights the importance of anammox as well as denitrification in driving ecosystem N losses, which is critical for improving the current global N cycle model and achieving sustainable N management.

## Graphical abstract

This work conducts a global synthesis including 3240 observations from 199 published isotope pairing studies and finds that denitrification governs microbial nitrogen loss in the form of N_2_ (79.8±0.4%) in both terrestrial and aquatic ecosystems. It is important to note that anammox is more important in aquatic than terrestrial ecosystems worldwide.

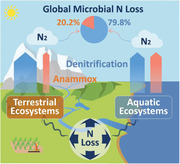

## 1 Introduction

Nitrogen (N) is a key element controlling the structure and functioning of terrestrial and aquatic ecosystems.^[^ ^1^ ^]^ However, over the past century, N fertilization has substantially increased the N inputs to the Earth's surface.^[^ ^2^ ^]^ Human activities are estimated to contribute over 210 Tg N to global terrestrial ecosystems every year, of which approximately 70–100 Tg eventually enters coastal waters and the open ocean via river transport and atmospheric deposition.^[^ ^3^ ^]^ N overfertilization has resulted in multiple environmental issues, including water acidification, eutrophication, harmful algal blooms, and biodiversity reduction.^[^ ^2^ ^,^ ^4^ ^]^ A large amount of this N goes back to the atmosphere from terrestrial and aquatic ecosystems after being processed by microorganisms involved in denitrification and anaerobic ammonium oxidation (anammox) processes. While denitrification can reduce nitrate (NO_3_^−^) and nitrite (NO_2_^−^) to gaseous N under anaerobic conditions,^[^ ^5^, ^6^ ^]^ the anammox process combines ammonia (NH_4_^+^) and NO_2_^−^ to produce nitrogen gas (N_2_). Denitrification was regarded as the process behind N losses before the discovery of the anammox process in 1995.^[^ ^7^ ^]^ However, Devol^[^ ^8^ ^]^ showed that anammox accounted for 0–79% of total N_2_ production in the marine environment and dominated over denitrification in deep‐sea habitats. Until now, the relative contribution of denitrification (rd) and anammox (ra) to N loss across contrasting ecosystems worldwide has been unknown. This lack of knowledge hampers our ability to understand how and why N is being lost to the atmosphere, with the consequences for predicting climate change and land use intensification impacts on global N cycling.

Three main aspects have limited our understanding of the relative contribution of denitrification and anammox processes to global N losses. First, most studies investigating the influence of denitrification and anammox processes on N losses have been conducted at the local scale and within particular ecosystems (e.g., soil, sediment, or water), but a large‐scale study investigating the global biogeography and contribution of denitrification and anammox to N losses is largely lacking. Second, we lack a comprehensive understanding of the environmental drivers explaining both denitrification and anammox processes across global environmental gradients and contrasting ecosystems. Denitrification and anammox processes are known to respond differently to changes in a number of environmental and biological factors, such as temperature, redox condition, N content, organic matter availability, and microorganisms.^[^ ^9^, ^10^ ^]^ Therefore, differences in the relative importance of denitrification and anammox in N loss may be environmental context‐dependent. However, empirical evidence is still limited. Finally, the different contributions of denitrification and anammox to N loss are commonly associated with the competitive capacity of denitrifying and anammox bacteria for NO_2_^−^, which is the common substrate for the two processes.^[^ ^11^ ^]^ Nevertheless, a mechanism understanding, based on isotopic approaches, of the relative contributions of denitrification and anammox to N loss on a global scale is still missing.

Here, we compiled a global dataset comprising 3240 observations of denitrification and anammox from 199 published ^15^N isotope pairing studies and conducted a meta‐analysis. The objectives were to (1) reveal the global pattern of the contribution of denitrification and anammox processes to N losses in the form of N_2_ across contrasting environments (i.e., water, sediment, and soil), (2) explore the spatial variation in the contribution of denitrification and anammox to N loss, and (3) determine the key factors influencing the contribution of denitrification and anammox to N loss worldwide. By addressing this knowledge gap, we aim to advance our knowledge on the importance of denitrification and anammox to N loss worldwide and further create the first global atlas of the contribution of these microbial processes to ecosystem N losses.

## 2 Results

### 2.1 Global Pattern of the Contribution of Denitrification and Anammox to Microbial Nitrogen Loss

**Figure 1:**
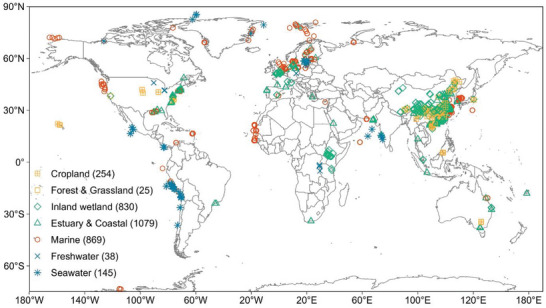
Global distribution of field data on the relative contributions of denitrification and anammox to nitrogen loss. These data consisted of 3240 observations from 199 experimental studies and were grouped into seven ecosystems. The investigated environmental matrices included terrestrial soil (yellow symbols), wetland sediment (green symbols), marine sediment (red symbols), and water (blue symbols). Numbers in parentheses are the number of observations in each ecosystem.

**Figure 2:**
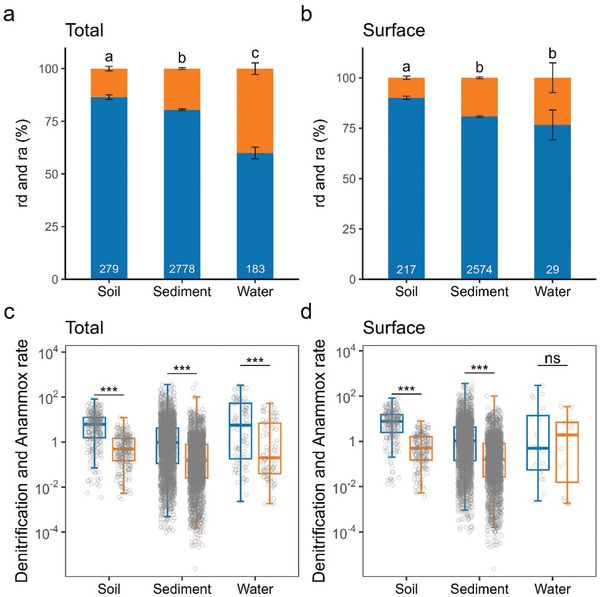
Global patterns of the relative contributions of denitrification (rd) and anammox (ra) to nitrogen loss in different environmental matrices. a,b) The rd (blue) and ra (yellow) in total (a) and surface (b) (0–0.2 m for soil and sediment, 0–20 m for water) soil, sediment, and water. The bar is the standard error, the different letters above bars mean significant differences (*p* < 0.05), and the numbers at the bottom are the number of observations in each environment. c,d) The comparison of denitrification rates (blue) and anammox rates (yellow) in total (c) and surface (d) environments. Units: nmol g^−1^ h^−1^ for soil and sediment, µmol m^−3^ h^−1^ for water. Significance: ***p < 0.001; **p < 0.01; *p < 0.05; ns, *p* > 0.05.

Through a synthesis of all available data from published studies across the main aquatic and terrestrial ecosystems (**Figure** **1**), we found that denitrification dominated the microbial N losses compared with anammox at the global scale (**Figure** **2**a). The average contribution of denitrification to N losses (mean ± standard error) was 86.5 ± 1.1% in soil, 80.4 ± 0.4% in sediment, and 59.9 ± 2.8% in water (Figure 2a), suggesting that denitrification is more important in terrestrial than in aquatic ecosystems worldwide. The results were consistent with the fact that the denitrification rates were clearly higher than anammox rates (Figure 2c; Table S1, Supporting Information). Similar results were also observed in surface soils, sediment (0–0.2 m), and water (0–20 m) (Figure 2b,d). Additionally, the contribution of denitrification and anammox to N loss varied across different ecosystems (Figure S1, Supporting Information). In inland wetlands, the contribution of anammox in sediment (27.4 ± 1.0%) was significantly greater compared to terrestrial and other aquatic ecosystems. It was noteworthy that anammox could contribute up to 43.2% and 28.4% of N loss in global seawater and freshwater, respectively (Figure S1, Supporting Information).

### 2.2 Spatial Variation in the Contribution of Denitrification and Anammox to Microbial Nitrogen Loss

At the global scale, the contribution of denitrification and anammox to sediment N loss changed significantly with longitude (*p* < 0.05; Figures S2b,S3b, Supporting Information). In addition, the contribution of denitrification to N loss in soil and sediment was the lowest at the equator and increased toward the poles, and that of anammox was quite the contrary (Figures S2d,e, and S3d,e, Supporting Information). However, the contribution of denitrification to N loss in surface water decreased significantly with latitude (Figure S3f, Supporting Information). In vertical profiles, the contribution of denitrification to the N loss was lower in deep soil (>1 m) and sediment (0.6–1 m), while that of anammox was higher (Figure S4a, Supporting Information). Water depth had an inconsistent effect in freshwater and seawater (Figure S4a, Supporting Information). In freshwater, the contribution of denitrification and anammox did not vary with depth, whereas the contribution of anammox increased with increasing water depth in seawater.

### 2.3 Factors Influencing the Contributions of Denitrification and Anammox to Microbial Nitrogen Loss

Mean annual precipitation (MAP) and mean annual temperature (MAT) were two important factors affecting the contribution of denitrification and anammox to N loss in soil and sediment (Figures S5a,b, S6a,b, Supporting Information). Besides climatic factors, the contribution of denitrification to soil N loss was positively correlated with soil pH, density, total organic carbon (TOC) content, and the ratio of total carbon content and total nitrogen content (C: N ratio) (Figure S5c—f, Supporting Information) but negatively related to soil NO_3_^−^ and NH_4_^+^ contents (Figure S5g,h, Supporting Information). In the sediment, the contribution of denitrification to N loss was influenced by sediment properties and water parameters, such as sediment temperature, pH, TOC, and water depth (Figures S6,S7, Supporting Information). In freshwater and seawater, the contribution of denitrification to N loss decreased with increasing water depth and NO_3_^−^ content (Figure S8, Supporting Information). Among microbial variables, *nirS* gene abundance was positively related to the contribution of denitrification to soil N loss, and the abundances of *hzsB*, bacterial, and anammox 16S rDNA genes were positively related to the contribution of anammox (Figure S9, Supporting Information). Notably, the main factors influencing the contribution of denitrification and anammox to N loss varied across ecosystem types (Figures S10–S12, Supporting Information).

**Figure 3:**
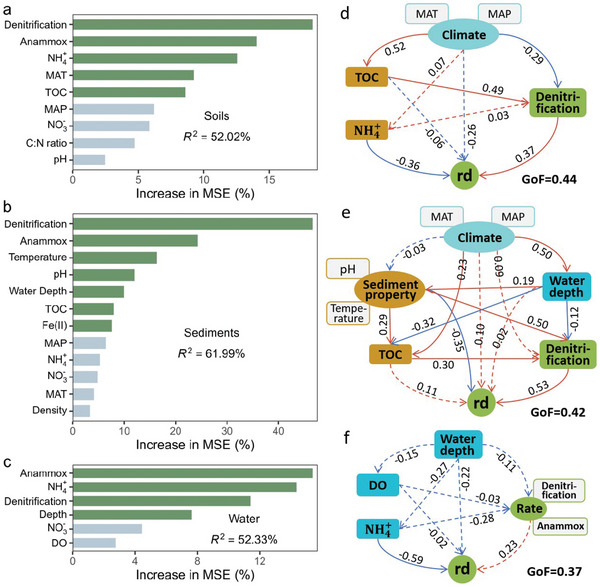
Drivers of the relative contributions of denitrification (rd) and anammox (ra) to nitrogen loss in different environmental matrices. a–c) The random forest model reveals the predictive importance of the geographic, climatic, and environmental factors for the contribution of denitrification and anammox in soils (a), sediments (b), and water (c). Green bars represent factors that are significant at *p* < 0.05. d–f) Partial least square path model (PLS‐PM) shows the direct and indirect effects of environmental variables on the contribution of denitrification (rd) in soils (d), sediments (e), and water (f). Numbers adjacent to arrows indicate the path coefficients. The red line indicates a positive correlation, and the blue line indicates a negative correlation. The solid line indicates that the effect is significant (*p* < 0.05). Abbreviations: MAT, mean annual temperature; MAP, mean annual precipitation; TOC, total organic carbon content; C: N ratio, the ratio of total carbon content and total nitrogen content; NO_3_^−^, nitrate content; NH_4_^+^, ammonia content; DO, water dissolved oxygen content.

Random forest analysis showed that in addition to denitrification and anammox rates, MAT, soil NH_4_^+^, and TOC were the main factors controlling the contribution of denitrification to soil N loss globally (**Figure** **3**a). In the sediment, the contribution of denitrification to N loss was largely controlled by sediment properties (i.e., temperature, pH, ferrous (Fe(II)), and TOC) and water depth (Figure 3b). However, the contribution of denitrification in the water was primarily determined by water depth and water NH_4_^+^ content (Figure 3c). The partial least squares‐path model (PLS‐PM) showed that climate regulated the contribution of denitrification to soil N loss indirectly through its effect on soil properties and process rates (Figure 3d). In aquatic environments, the influence of water depth on the contribution of denitrification was mainly mediated through sediment properties and water quality (Figure 3e,f).

## 3 Discussion

### 3.1 Denitrification Governs Terrestrial Nitrogen Losses at the Global Scale

Since its first discovery in 1995 in a wastewater treatment system,^[^ ^7^ ^]^ anammox has been frequently highlighted as a fundamental player in microbial N loss in a wide range of environments.^[^ ^9^ ^,^ ^12^ ^]^ However, our synthesis provided solid evidence that denitrification governs N losses in terrestrial ecosystems compared with anammox at the global scale (Figure 2a,b; Figure S1, Supporting Information). The relative contribution of denitrification and anammox differed significantly among the studied environments, with the contribution of denitrification decreasing in the order of soil > sediment > water (Figure 2). This result is expected because anammox bacteria are autotrophic, slow‐growing, and environmentally sensitive microorganisms and may be less competitive for NO_2_^−^ as the electron acceptor than heterotrophic denitrifying bacteria.^[^ ^13^ ^]^ In addition, anaerobic environments (e.g., impermeable soils) may prevent nitrification capable of providing abundant NO_2_^−^ to anammox microorganisms. Therefore, denitrification can be most important in environments with large amounts of labile organic carbon or an excess of carbon relative to N inputs.^[^ ^5^ ^]^

Uncertainties may exist in this global synthesis because the ^15^N isotope pairing technique has several potential limitations and drawbacks. First, the dissimilatory nitrate reduction to ammonium (DNRA), which also occurs under anaerobic or anoxic environments, can transform ^15^NO_3_^−^ to ^15^NH_4_^+^ when using ^15^NO_3_^−^ in isotope experiments. The ^30^N_2_ produced by the coupled DNRA‐anammox in the majority of previous studies is attributed to the denitrification process, probably leading to an overestimation of actual denitrification and an underestimation of anammox.^[^ ^14^ ^]^ However, this estimation error can be neglected when DNRA is not significant or when the incubation systems contain relatively high concentrations of ^14^NH_4_^+^.^[^ ^15^ ^]^ Additionally, some studies in our collection have corrected this error by calculating the mole fraction of ^15^NH_4_^+^ in total NH_4_^+^.^[^ ^16^, ^17^ ^]^ Second, the isotope pairing technique in slurry incubations generally does not consider the coupled nitrification‐denitrification process and thus may underestimate the contribution of denitrification to N loss, especially in the plant rhizosphere.^[^ ^6^ ^]^ Nevertheless, the isotope pairing technique included in this study is currently the best approach and perhaps the only reliable method to discriminate denitrification and anammox processes and can be effectively used to compare sites and experimental treatments and to assess controlling factors in terrestrial and aquatic ecosystems.^[^ ^18^ ^]^ In addition, field measurements in marine ecosystems have frequently been conducted in continental shelf areas. Therefore, it is important to note that our conclusions regarding marine N losses should be applied cautiously to the global oceans. Similarly, data for the freshwater column remain limited, highlighting the need for additional field measurements to enable a more accurate assessment.

### 3.2 Anammox Plays an Important Role in Seawater and Freshwater

Our synthesis showed that anammox could contribute up to 43.2% and 28.4% of N loss in global seawater and freshwater, respectively (Figure S1, Supporting Information), suggesting that anammox plays a relatively more minor but non‐negligible role in water N removal at the global scale. However, the contribution of anammox to water N removal is highly variable, ranging from 0 to as high as 100%. For example, anammox activity in the water column was not measurable at almost all depths of Lake Kivu, a deep meromictic tropical lake in East Africa.^[^ ^19^ ^]^ In the suboxic zone (85–115 m) of the central Black Sea, water‐column denitrification was not detected, and the anammox process was possibly the only source of N_2_ in seawater.^[^ ^20^ ^]^ Considering that nearly 90% of the ocean is deeper than 1000 m and hypoxia is expected in the oceans, especially in the bottom water column, Dalsgaard et al^[^ ^21^ ^]^ estimated that anammox was responsible for about 1/3 or even 1/2 of the global N loss from marine environments. Such estimates were close to the value obtained by our global synthesis (Figure S1, Supporting Information). The anammox process does not lead to nitrous oxide (N_2_O), a potent greenhouse gas that can be produced during incomplete denitrification.^[^ ^22^ ^]^ Therefore, the increased contribution of anammox to microbial N loss in aquatic ecosystems implies a decrease in N_2_O emissions from denitrification and therefore is of substantial significance in the context of climate warming.

### 3.3 Contrasting Factors Explain the Contribution of Denitrification and Anammox to Nitrogen Loss Worldwide

We found that the contribution of denitrification to soil and sediment N loss increased significantly from low to high latitude (Figure S2d,e, Supporting Information), similar to previous studies.^[^ ^23^ ^]^ The influences of latitude on the contribution of denitrification and anammox are mediated through its effects on climate and local environmental factors (Figures S13,S14, Supporting Information). Temperature and rainfall are key local factors regulating microbially mediated N cycling processes.^[^ ^18^ ^]^ Our results showed that the contribution of denitrification in soils declined significantly with MAT and MAP (Figure S5a,b, Supporting Information). These results suggested that a higher contribution of denitrification to terrestrial N loss will be detected generally in colder and drier areas, probably because denitrification rates increased less with MAT and MAP than anammox rates.^[^ ^23^, ^24^ ^]^ In addition, a lower contribution of denitrification to N loss was detected in the deep soils and sediments (Figure S4a, Supporting Information). Such results might be attributed mainly to the significant decrease in N and organic carbon availability with increasing depth (Figures S13,S14, Supporting Information). As organic carbon is the primary electron donor for denitrifiers, the lower TOC content in the deep soils and sediments generally resulted in lower denitrification rates^[^ ^25^ ^]^ and a lower contribution of denitrification to N loss. However, anammox metabolism is autotrophic, using carbon dioxide (CO_2_) as the main carbon source, and therefore can tolerate oligotrophic environments.^[^ ^26^ ^]^ In addition, a lower soil C: N ratio generally results in high anammox rates but does not affect denitrification rates,^[^ ^24^ ^]^ resulting in denitrification contributing less to N loss than anammox in deep soils and sediments.

Among soil and sediment properties, pH is one of the most important factors explaining the spatial variation of biogeochemical processes.^[^ ^27^ ^]^ We found that the contribution of denitrification was also positively correlated with soil pH (Figure S5c, Supporting Information), which may be attributed to the fact that higher pH can alleviate N limitation to microbes and thereby enhance the denitrification rate^[^ ^24^ ^,^ ^28^ ^]^ but had no influence on anammox rate.^[^ ^23^ ^]^ The results also indicated that anammox might contribute more in extremely acidic soil environments. The contribution of denitrification and anammox to sediment N loss had a contrary relationship with sediment pH (Figure S6d, Supporting Information), which may be attributed to an increase in sediment C: N ratio with increasing pH in our database (Figure S14, Supporting Information). High NH_4_^+^ content normally benefits anammox metabolism,^[^ ^29^ ^]^ causing a higher contribution of anammox to soil N losses (Figure S5 h, Supporting Information). However, this was not true in coastal and marine sediments because of the lack of light and oxygen,^[^ ^30^ ^]^ accompanied by NH_4_^+^ accumulation (Figure S11, Supporting Information). Iron is critical to most redox enzymes involved in microbial respiration.^[^ ^30^ ^]^ Fe(II) is typically more soluble and, therefore, more bioavailable.^[^ ^31^ ^]^ Fe(II) oxidation generally couples to the reduction of NO_3_^−^ to N_2_ or to NH_4_^+^ under anoxic conditions.^[^ ^31^ ^]^ In this study, higher Fe(II) content resulted in a higher contribution of denitrification to marine sediment N loss (Figure S11, Supporting Information). Also, anammox bacteria can use Fe (II) as electron donors.^[^ ^32^ ^]^ The oxic‐anoxic interfaces like riparian and coastal sediments are normally favorable for iron redox and anammox.^[^ ^31^ ^,^ ^33^ ^]^ Thus, higher sediment Fe(II) content could lead to a higher contribution of anammox to estuary and coastal sediment N loss (Figure S11, Supporting Information). Bacteria containing *nirS* gene can produce copper‐containing nitrite reductase, thereby enhancing the reduction of NO_2_^‐^ to nitric oxide (NO), the rate‐limiting step in denitrification.^[^ ^32^ ^]^ Higher *nirS* gene abundance generally implies stronger denitrification activity, causing a higher contribution to soil N loss (Figure S9c, Supporting Information). Hydrazine synthase catalyzes the reaction of NH_4_^+^ and NO_2_^−^ to form hydrazine as a free intermediate in anammox processes and is encoded by *hzsA* and *hzsB* genes.^[^ ^32^ ^]^ Increasing *hzsB* gene abundance can boost the anammox rate^[^ ^23^ ^]^ and the contribution of anammox to N loss (Figure S9d, Supporting Information).

Regarding water denitrification and anammox, this study found that water depth was one of the most critical environmental factors controlling the contribution of denitrification and anammox to N_2_ production (Figure 3c). A previous synthesis of anammox studies in the marine environment indicated that water depth was a critical factor controlling the relative importance of denitrification and anammox in N loss, with the anammox process theoretically producing 29% of the N_2_.^[^ ^34^ ^]^ In general, low availability of organic carbon and dissolved oxygen, high levels of inorganic N, and warm water temperature have been considered to enhance the contribution of anammox to N removal.^[^ ^35^ ^]^ Our synthesis confirmed that the contribution of anammox in seawater was positively correlated with water temperature and negatively correlated with dissolved oxygen availability (Figure S12, Supporting Information), both of which were negatively affected by water depth (Figure S15, Supporting Information). A previous study also reported that the contribution of anammox to the total N_2_ production increased with water depth from 17% at the shelf to 57% at the basin in the East Sea, mainly due to an increased NO_2_^−^ availability for anammox.^[^ ^36^ ^]^ Overall, our results emphasize that water depth is important in regulating the relative importance of denitrification and anammox to water N loss, and the environmental changes caused by water depth can be the proximal controls of the contribution of anammox and denitrification.

### 3.4 The Implications for Nitrogen Retention or Removal in Terrestrial and Aquatic Ecosystems

**Figure 4:**
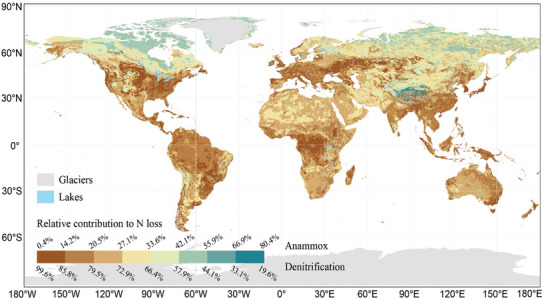
Global spatial variations in the proportion of the relative contributions of denitrification and anammox to nitrogen loss from terrestrial soils. The prediction was achieved using global climatic and surface edaphic (0–0.2 m) variables.

**Figure 5:**
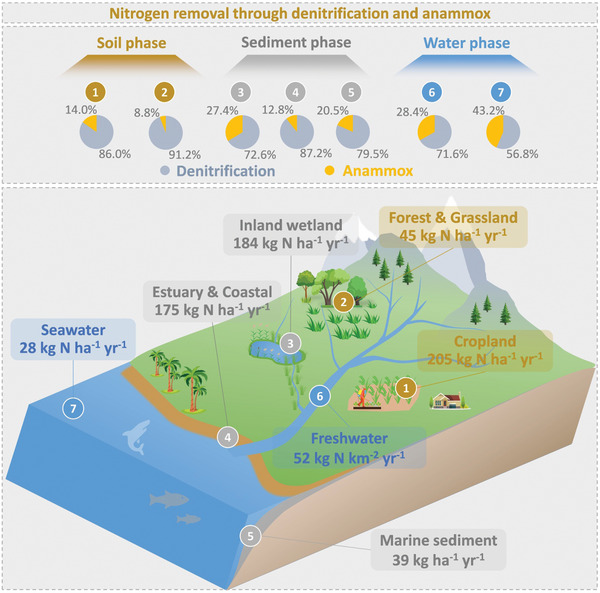
Annual contribution of denitrification and anammox to nitrogen losses in different ecosystems. The values were estimated based on the average rate of denitrification and anammox in each ecosystem.

In terrestrial ecosystems such as croplands and forests, N is a key nutrient controlling plant productivity.^[^ ^1^ ^]^ However, this study found that denitrification can result in a significant N loss, contributing 86.5% of the microbial N loss in global soils (Figure 2a), and therefore deserves more attention from the perspective of maintaining soil fertility. According to our prediction model, the highest contribution of denitrification to N loss was mainly found in the northern Great Plains of North America, the Eastern European Plain, the Brazilian Plateau, and the Congo Basin (**Figure** **4**), which coincides with the global distribution of croplands and forests.^[^ ^37^ ^]^ N loss through denitrification from terrestrial ecosystems was approximately 217 kg N ha^−1^ yr^−1^, of which 81.1% occurred in agricultural soils (**Figure** **5**). To reduce the denitrification N loss in terrestrial ecosystems, especially in croplands, numerous management practices have been applied, including the use of NH_4_^+^‐based fertilizers, decreasing N application rate, biochar amendment, use of nitrification inhibitor, and advanced irrigation techniques.^[^ ^38^, ^39^ ^]^ Besides causing substantial N loss, another key concern is that the denitrification process can produce a certain amount of N_2_O when the reaction is incomplete.^[^ ^40^ ^]^ A recent study estimated that approximately 8% of denitrified N was converted to N_2_O in terrestrial ecosystems at the global scale.^[^ ^41^ ^]^ Although N_2_O accounts for only 0.03% of global greenhouse gas emissions, it has a global warming potential of 298 times greater than that of CO_2_ and contributes to stratospheric ozone destruction.^[^ ^40^ ^]^ Therefore, more attention should be paid to not only regulating the amount of total denitrification but also facilitating the reduction of N_2_O to N_2_ during denitrification and reducing N_2_O emissions from terrestrial ecosystems.

In aquatic ecosystems, particularly in eutrophic waters, the microbial N removal processes are extremely important because excess N has caused a number of environmental problems.^[^ ^4^ ^]^ Our global synthesis showed that both denitrification and anammox processes played a critical role in mitigating the water N pollution, contributing 59.9% and 40.1% of the microbial N loss, respectively (Figure 2). Significantly, aquatic ecosystems are a major source of N_2_O to the atmosphere, contributing a third (5.4 Tg N yr^−1^) of global anthropogenic N_2_O emission,^[^ ^42^ ^]^ and denitrification is the largest contributor to N_2_O emission. For example, Ji et al. estimated that denitrification contributed to approximately 20% of global oceanic N_2_O production.^[^ ^43^ ^]^ Beaulieu et al. found that denitrification of water NO_3_^−^ accounted for 26% of N_2_O emissions in streams and rivers.^[^ ^44^ ^]^ However, compared to denitrification, the anammox process does not lead to N_2_O emission. In addition, anammox could reduce N_2_O production by consuming NH_4_^+^ and NO_2_^−^ that might otherwise follow the N_2_O‐generating pathways, including denitrification and nitrification.^[^ ^45^ ^]^ Therefore, although anammox bacteria have a slow growth rate and are sensitive to environmental changes, the anammox process is of great interest in water N pollutant removal.^[^ ^40^ ^]^ Several methods have been used to improve the stability of the anammox process and increase the anammox rates, especially in wastewater treatment, including increasing the concentration of anammox microorganisms and modifying reactor configuration.^[^ ^46^ ^]^

## 4 Conclusions

Our synthesis allows us to assess, for the first time, the relative importance of denitrification and anammox in microbial N loss at the global scale and reveals that denitrification accounts for 86.5%, 80.4%, and 59.9% of the total N_2_ production in soil, sediment, and water, respectively. We demonstrate that variations in the contribution of denitrification to microbial N loss can be explained by both large‐ and local‐scale factors. On a global scale, the contribution of denitrification to N loss increased significantly with increasing latitude. Temperature and precipitation may mediate such geographic patterns in soils, and water depth and sediment temperature may mediate such patterns in sediments. In both soil and sediment, depth, pH, and TOC are key local‐scale factors determining the contribution of denitrification to N loss. Water depth is important in regulating the relative contribution of denitrification and anammox to water N loss in the water column. Overall, our study fills a critical knowledge gap regarding the global patterns of N loss and highlights the importance of both denitrification and anammox in explaining ecosystem N losses worldwide, which is critical for predicting the impacts of human alterations on the global N cycle.

## 5 Experimental Section

### Data Collection

All peer‐reviewed publications were searched in the Web of Science database and Google Scholar before August 2022. The following term combinations were used for the search in title, keyword, or abstract: denitrif* AND (anammox OR “anaerobic ammonia oxidation” OR “anaerobic ammonium oxidation”). The publication sources cited in the earlier reviews were also gathered and reevaluated. The following criteria were used to select eligible studies: (1) the rates of denitrification and anammox were measured using isotope pairing technique; (2) both rates or at least one of their contributions to N loss in the form of N_2_ were reported or could be calculated from the information provided in the publication; (3) the data from constructed wetland and sewage treatment plant, were excluded from this dataset due to human intervention and high nutrient inputs. Based on these criteria, 199 studies were retained for further analysis.

Site‐specific information was also extracted from the original articles, including the geographic coordinates (i.e., latitude and longitude of experimental sites), climatic variables (e.g., MAT and MAP), ecosystem types, physicochemical properties of soil and sediment (e.g., the contents of sand/clay, pH, moisture content), water physicochemical properties (e.g., depth, pH, salinity, oxygen content), C and N pools (e.g., TOC, total N, dissolved organic carbon, NH_4_^+^, NO_3_^−^, NO_2_^−^, and C: N ratio), microbial variables (e.g., the abundance of *nirS, nirK*, and *hzsB*). When MAT and MAP were not available in articles, they were obtained from the global climatic database through latitude and longitude. If a study reported organic matter concentration, TOC concentration was calculated using a conversion factor of 0.58.^[^ ^47^ ^]^ The missing values of the relative contribution of denitrification and anammox to N loss were calculated through their rates. The data presented as graphs were extracted with WebPlotDigitizer version 4.4. Finally, 3240 pairs of global data on the relative contributions of denitrification and anammox were obtained (Figure 1).

### Data Analysis

Denitrification and anammox rates were standardized to µmol N m⁻^3^ h⁻¹ for water and nmol N g⁻¹ h⁻¹ for soil and sediment, accounting for sampling depth and substrate density. The denitrification rate was adjusted to 25 °C using a fixed value (2.40) of *Q_10_* to avoid measurement errors due to different laboratory incubation temperatures.^[^ ^18^ ^]^ The equation for standardization was:

(1) $$N_{den1}/N_{den2}={Q_{10}}^{\left(25-T1\right)/10}$$

Where *N*_den1_ was the original denitrification rate, and *N*_den2_ was the recalibrated denitrification rate at 25 °C. *T*_1_ was the incubation temperature for *N*_den1_.

The anammox rate was also adjusted to 25 °C using a fixed value of *Q_10_*, 2.15.^[^ ^18^ ^]^ The equation was:

(2) $$N_{amx1}/N_{amx2}={Q_{10}}^{\left(25-T1\right)/10}$$

Where *N*_amx1_ was the original anammox rate, and *N*_amx2_ was the recalibrated anammox rate at 25 °C. *T*_1_ was the incubation temperature for *N*_amx1_.

To simplify the analysis, some ecosystem types with similar characteristics were combined. Shallow (20–200 m) and deep‐sea (>200 m) benthic environments were classified as marine ecosystems, while estuarine and coastal wetlands, lagoons, and benthic environments (<20 m) were classified as estuarine and coastal ecosystems. Riparian zones and benthic environments in rivers, lakes, ponds, and ditches were classified as inland wetland ecosystems. Due to limited data, forests and grasslands were combined into one group, and cropland does not distinguish between dry and paddy fields. The relative contributions of denitrification and anammox to total N loss were calculated for each type of ecosystem and environmental matrices. The differences across environmental matrices, ecosystem types, and depths were analyzed using the Kruskal‐Wallis test with Fisher's least significant difference comparison from the “*agricolae*” R package. Considering that the sample sizes declined with increasing depth, the depth of soil and sediment was divided by 0.2, 0.4, 0.6, and 1 m, and water depth was divided by littoral (<20 m), shallow (20–200 m), deep‐sea (>200 m) depth. Robust linear regression based on the MM estimator was used to examine the bivariable relationships between the relative contributions of denitrification and anammox and environmental factors using the “*robustbase*” R package. Spearman correlation analysis was conducted to explore the relationship between geographic, climatic, and environmental factors using the “*psych*” R package.

Random forest (RF) models were performed to confirm the relative importance of main factors (method: percentage increases in the mean squared error (MSE)) using the R package “*linkET*”. The partial least squares‐path model (PLS‐PM) was conducted to explore the multivariable relationships between the relative contributions of denitrification and anammox and environmental factors across different environmental matrics. In the PLS‐PM, the variability in the observed variables was adequately captured by their latent variables when the loadings were greater than 0.7.^[^ ^48^ ^]^ The model was performed using the “*plspm*” R package, and the model reliability was evaluated using the Goodness of Fit (GoF) statistic.^[^ ^49^ ^]^ Furthermore, the effects of environmental factors on the relative contributions of denitrification and anammox across different ecosystems were tested by the linear mixed model using the “*lme4*” R package. For each model, “study” was considered as the random effect to eliminate the same‐study effect, and replicates were used as weight. All statistical analyses were performed using R software (version 4.2.2).

### Global Prediction

Global patterns of the relative contributions of denitrification and anammox in terrestrial soils (0–0.2 m) were predicted using five machine learning algorithms: RF, support vector machine (svmRadial), k‐nearest neighbor (knn), stepwise regression (leapSeq), and generalized linear models (glmnet). Seven predictors were selected to create the models, including climatic factors (MAT and MAP) and soil attributes (pH, C, N, clay content, and bulk density) because they were the primary factors affecting the microbial community and N loss rates.^[^ ^24^, ^25^ ^,^ ^50^ ^]^ The “*caret*” and “*caretEnsemble*” packages were used to train the five prediction models.^[^ ^51^ ^]^ The global soil attributes data with a resolution of 1.0 km was collected from the Harmonized World Soil Database (version 2.0) of the Food and Agriculture Organization of the United Nations (FAO) (gaez.fao.org/pages/hwsd). Climatic data with a resolution of 1.0 km was downloaded from WorldClim (www.worldclim.org).^[^ ^52^ ^]^ The world terrestrial boundary data were downloaded from the Natural Earth Database (www.naturalearthdata.com). The worldwide distribution maps were created using ESRI ArcGIS 10.6 software. All predictions were performed using R software (version 4.2.2).

The predictions of global relative contributions of denitrification and anammox were evaluated using 10‐fold cross‐validation with five replications. The mean of the absolute value of errors (MAE), the root mean square error (RMSE), and the regression coefficients of determination (*R* ^2^) were selected as validation indicators. A 10‐fold cross‐validation randomly splits the training database into ten equal subsets, nine of which were used as training data and one as test data. The test results of each subsample were averaged to estimate the performance of the models. The model with the lowest MAE, lowest RMSE, and greatest *R* ^2^ was selected as the best. Ultimately, random forest outperformed other algorithms in predicting the relative contributions of denitrification and anammox, with the 10‐fold cross‐validation *R^2^* of 0.56 (Figure S16, Supporting Information).

## Conflict of Interest

The authors declare no conflict of interest.

## Author Contributions

G.H., D.D. contributed equally to this work. M.D.‐B., Q.Z., and W.L. developed the original idea of the analyses presented in the manuscript. G.H. and D.D. collected and analyzed the data. G.H., M.D.‐B., Q.Z., and W.L. wrote the original draft. M.D.‐B. and W.L. contributed significantly to improve subsequent drafts.

## Supporting information



Supporting Information

## Data Availability

The data that support the findings of this study are openly available in figshare at https://doi.org/10.6084/m9.figshare.23578044, reference number 23578044.
