# Supplementary material for: Global Relative Importance of Denitrification and Anammox in Microbial Nitrogen Loss Across Terrestrial and Aquatic Ecosystems
Source: Adv Sci (Weinh). 2024 Dec 31;12(8):2406857. doi: 10.1002/advs.202406857 (PMC11848586; doi:10.1002/advs.202406857)
Supplement: Supplementary file 1 — Supporting Information [file ADVS-12-2406857-s001.docx]

**Supplementary Information**

**Global Relative Importance of Denitrification and Anammox in Microbial Nitrogen Loss across Terrestrial and Aquatic Ecosystems**

*Gang He ^1^†, Danli Deng ^2^†, Manuel Delgado-Baquerizo ^3*^, Wenzhi Liu ^1, 4*^, Quanfa Zhang ^4^*

^1^ Hubei Key Laboratory of Wetland Evolution & Ecological Restoration, Wuhan Botanical Garden, Chinese Academy of Sciences, Wuhan 430074, P.R. China.

^2^ Hubei Field Observation and Scientific Research Stations for Water Ecosystem in Three Gorges Reservoir, China Three Gorges University, Yichang, 443002, P.R. China.

^3^ Laboratorio de Biodiversidad y Funcionamiento Ecosistémico. Instituto de Recursos Naturales y Agrobiología de Sevilla (IRNAS), CSIC, Av. Reina Mercedes 10, E-41012, Sevilla, Spain.

^4^ Danjiangkou Wetland Ecosystem Field Scientific Observation and Research Station, Chinese Academy of Sciences & Hubei Province, Wuhan, 430074, P.R. China.

The first two authors contributed equally to this work.

***Corresponding author**

Phone: +86 27 87700849

Fax: +86 27 87700877

Email: M.delgado.baquerizo@csic.es (Manuel Delgado-Baquerizo);

liuwz@wbgcas.cn (Wenzhi Liu)

**Table S1. The average rates (mean ± standard error) of denitrification and anammox in different environmental matrices.** The average rates were calculated based on total and surface (0-0.2 m for soil and sediment, 0-20 m for water) datasets, respectively.

| Datasets | Environmental matrices | Denitrification rate ^a)^ | Anammox rate |
| --- | --- | --- | --- |
| Total | Soil | 9.7 ± 0.7 | 0.9 ± 0.1 |
|  | Sediment | 6.2 ± 0.4 | 1.2 ± 0.2 |
|  | Water | 26.0 ± 5.1 | 3.7 ± 0.8 |
| Surface | Soil | 11.5 ± 0.9 | 0.9 ± 0.1 |
|  | Sediment | 6 ± 0.4 | 0.9 ± 0.1 |
|  | Water | 28.3 ± 13.7 | 4.1 ± 1.9 |

a) Rate units: nmol g^-1^ h^-1^ for soil and sediment, μmol m^-3^ h^-1^ for water.


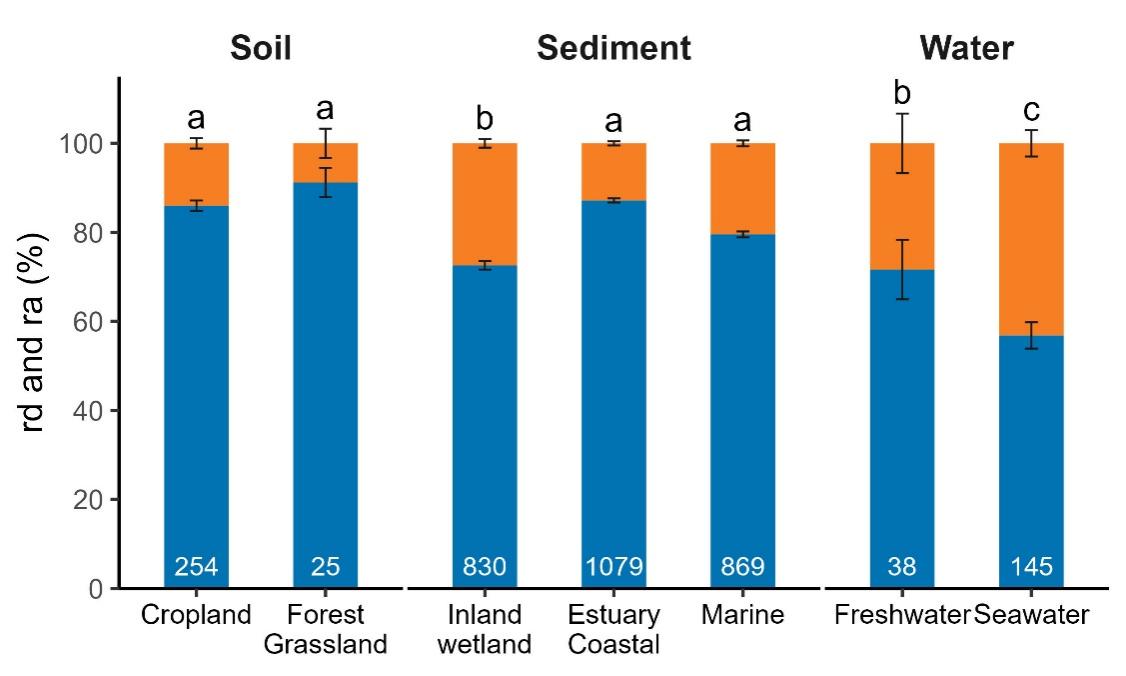


**Figure S1.** **The changes of the contribution of denitrification (rd, blue) and anammox (ra, yellow) with ecosystem types.** The numbers at the bottom are the number of observations in each ecosystem, the bar is the standard error, and the different letters above the bars mean significant differences (*p* < 0.05).


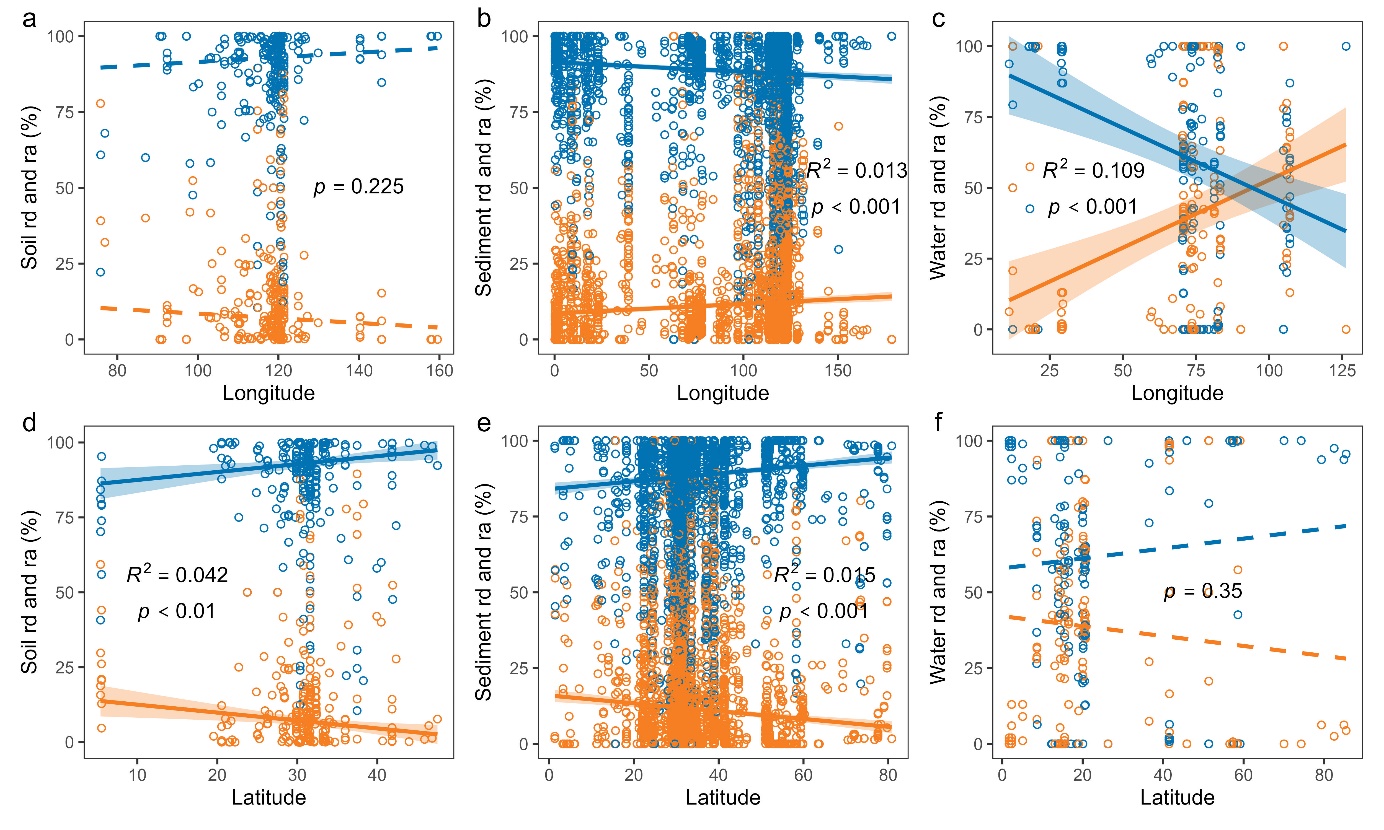


**Figure S2. The changes of the contribution of denitrification (rd, blue) and anammox (ra, yellow) with latitude and longitude in different environmental matrices. a-c**, The changes of the contribution of denitrification and anammox with longitude in soils (**a**), sediments (**b**), and water (**c**). **d-f**, The changes of the contribution of denitrification and anammox with latitude in soils (**d**), sediments (**e)**, and water (**f**). The linear regression models of both rd and ra had the same statistical test results.


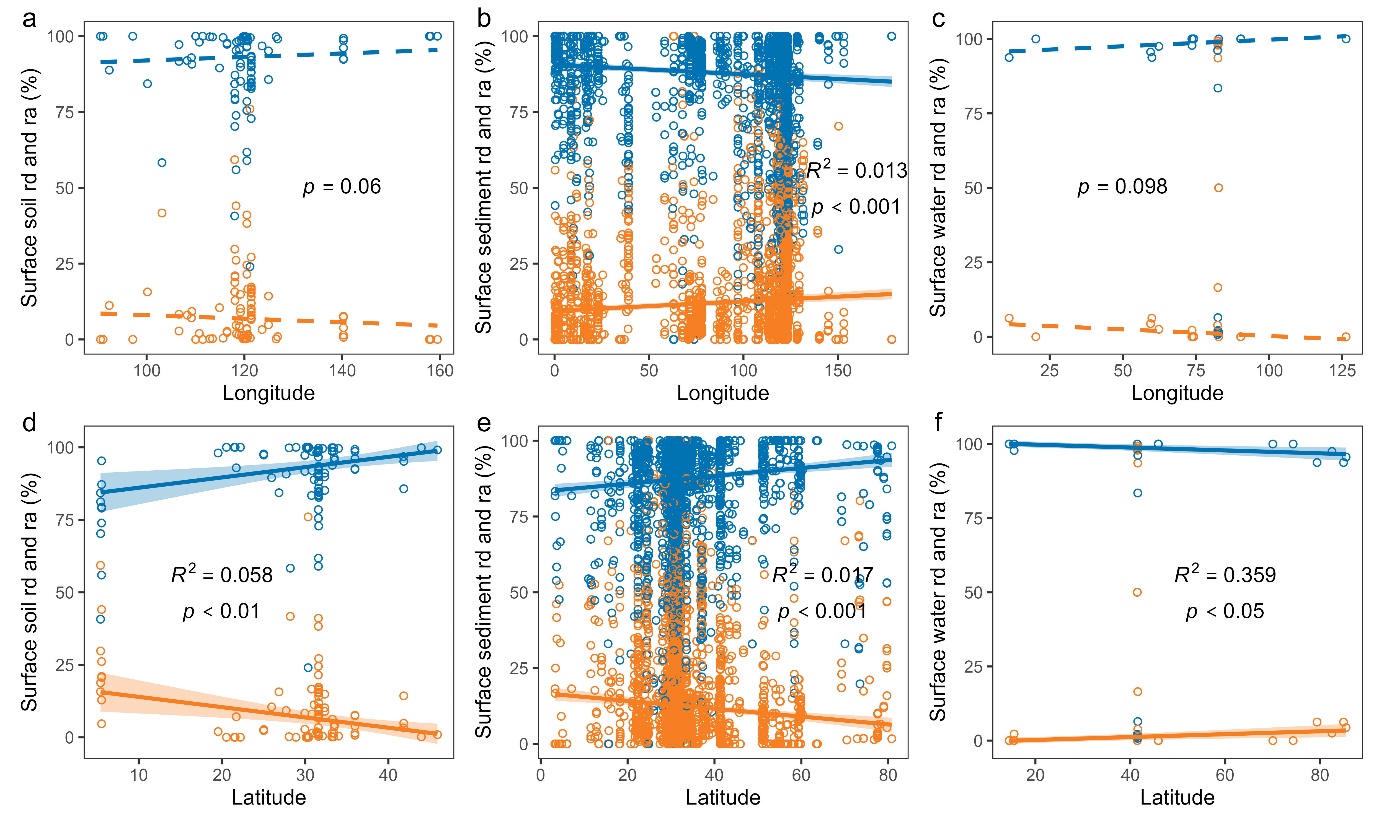


**Figure S3.** **The changes of the contribution of denitrification (rd, blue) and anammox (ra, yellow) with latitude and longitude in surface environmental matrices (0-0.2 m for soil and sediment, 0-20 m for water). a-c**, The changes of the contribution of denitrification and anammox with longitude in surface soils (**a**), sediments (**b**), and water (**c**). **d-f**, The changes of the contribution of denitrification and anammox with latitude in surface soils (**d**), sediments (**e)**, and water (**f**). The linear regression models of both rd and ra had the same statistical test results.


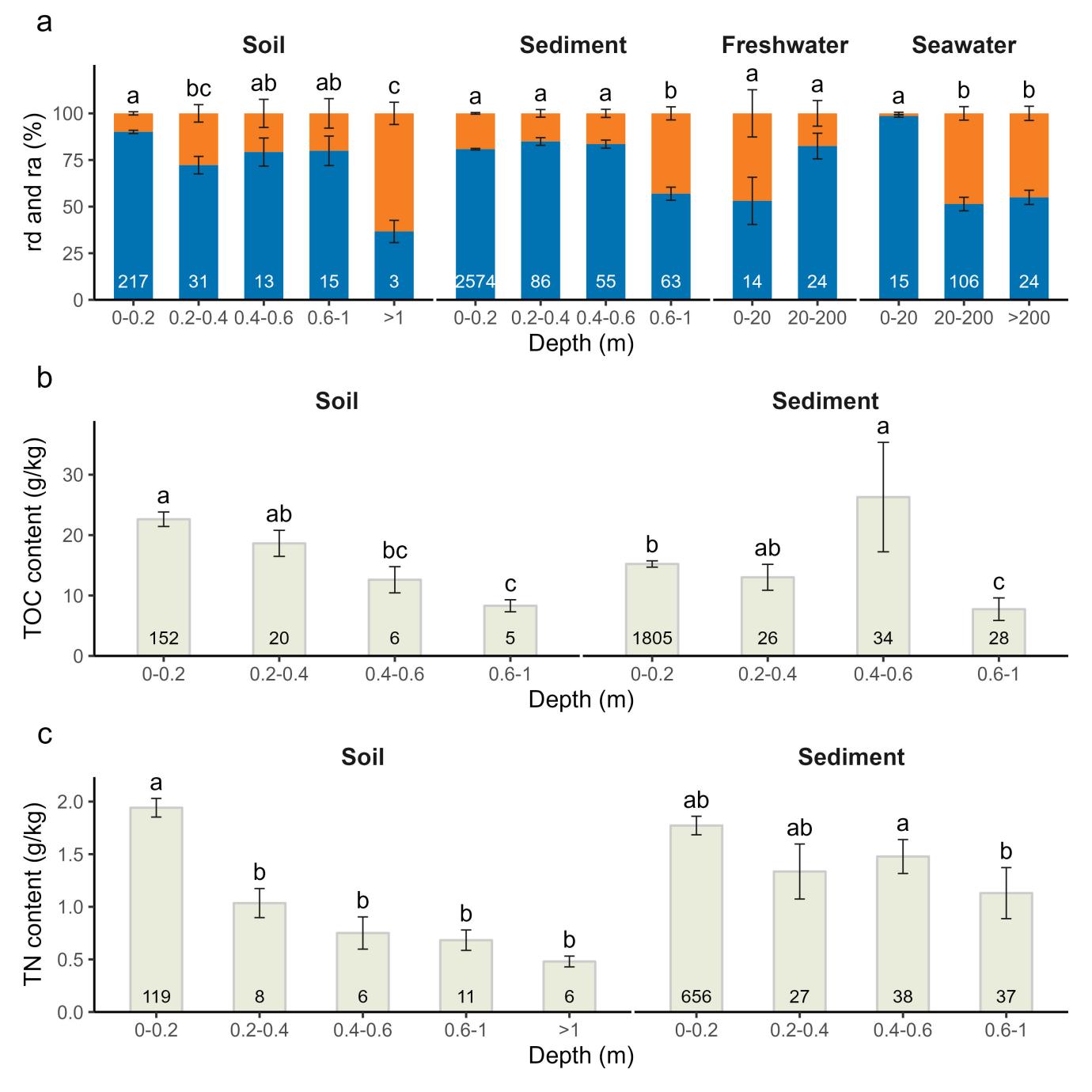


**Figure S4. The influence of depth on the contribution of denitrification (rd, blue) and anammox (ra, yellow) (a), total organic carbon content (TOC, b) and total nitrogen content (TN, c).** The numbers at the bottom are the number of observations in each depth and ecosystem type, the bar is the standard error, and the different letters above the bars mean significant differences (*p* < 0.05).


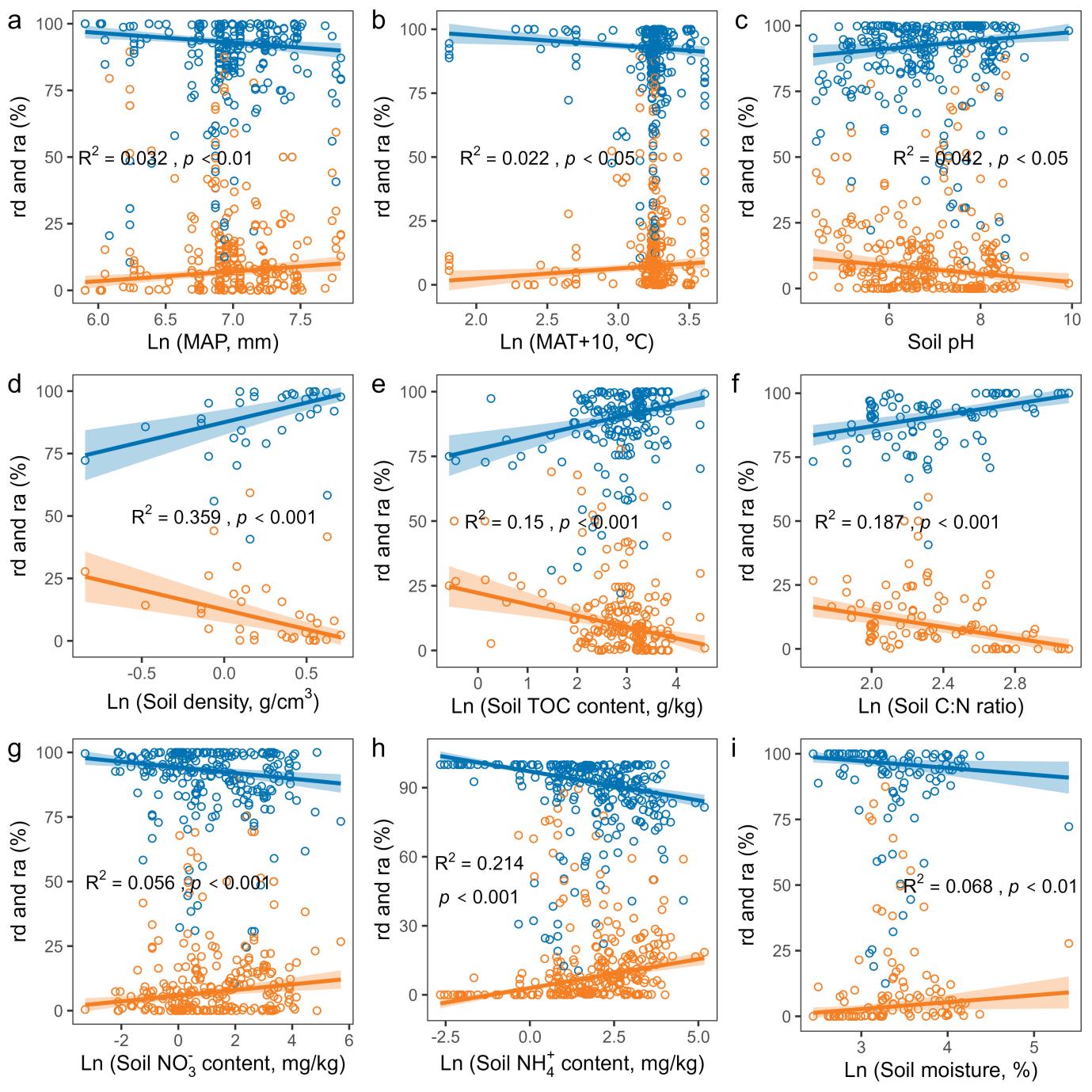


**Figure S5. Bivariate relationships between soil parameters and the contribution of denitrification (rd, blue) and anammox (ra, yellow) in soils.** The linear regression models of both rd and ra had the same statistical test results. Abbreviations: MAT, mean annual temperature; MAP, mean annual precipitation; TOC, total organic carbon; NO_3_^-^, nitrate; NH_4_^+^, ammonia; C:N ratio, ratio of total carbon content and total nitrogen content.


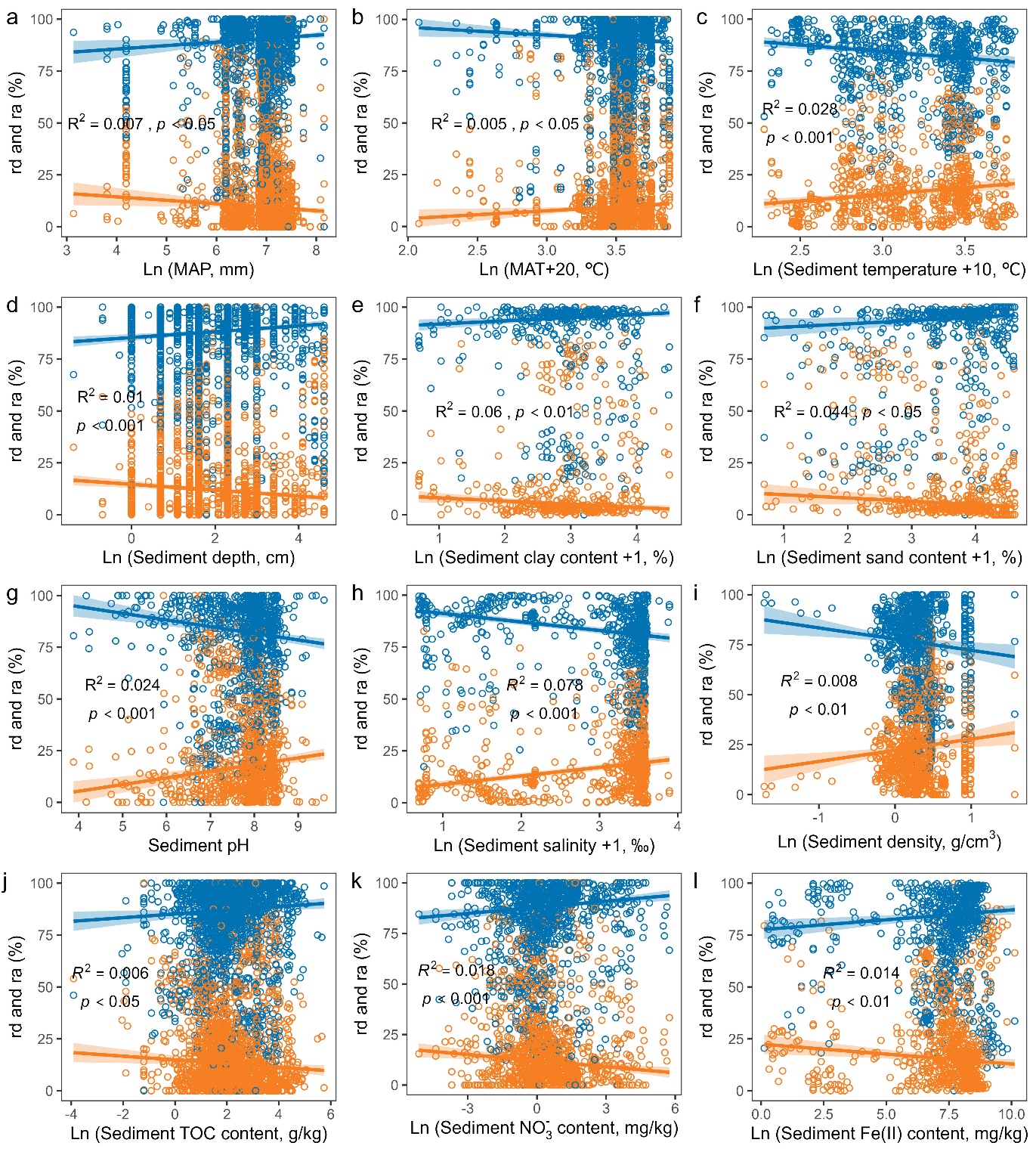


**Figure S6. Bivariate relationships between climatic factors and sediment parameters and the contribution of denitrification (rd, blue) and anammox (ra, yellow) in sediments.** The linear regression models of both rd and ra had the same statistical test results. Abbreviations: MAT, mean annual temperature; MAP, mean annual precipitation; TOC, total organic carbon; NO_3_^-^, nitrate; NH_4_^+^, ammonia; Fe(II), ferrous ion.


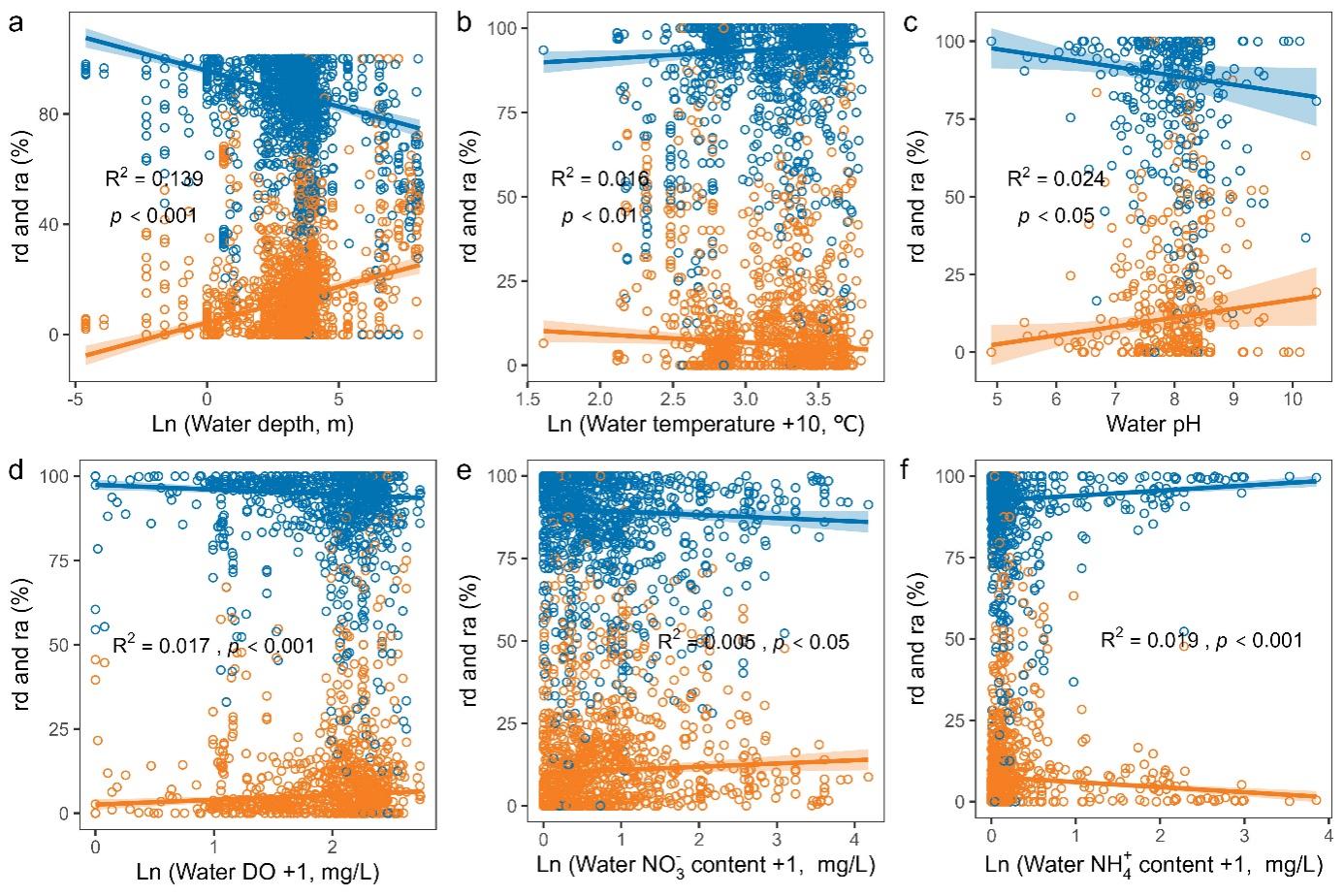


**Figure S7. Bivariate relationships between water parameters and the contribution of denitrification (rd, blue) and anammox (ra, yellow) in sediments.** The linear regression models of both rd and ra had the same statistical test results. Abbreviations: DO, dissolved oxygen; NO_3_^-^, nitrate; NH_4_^+^, ammonia.


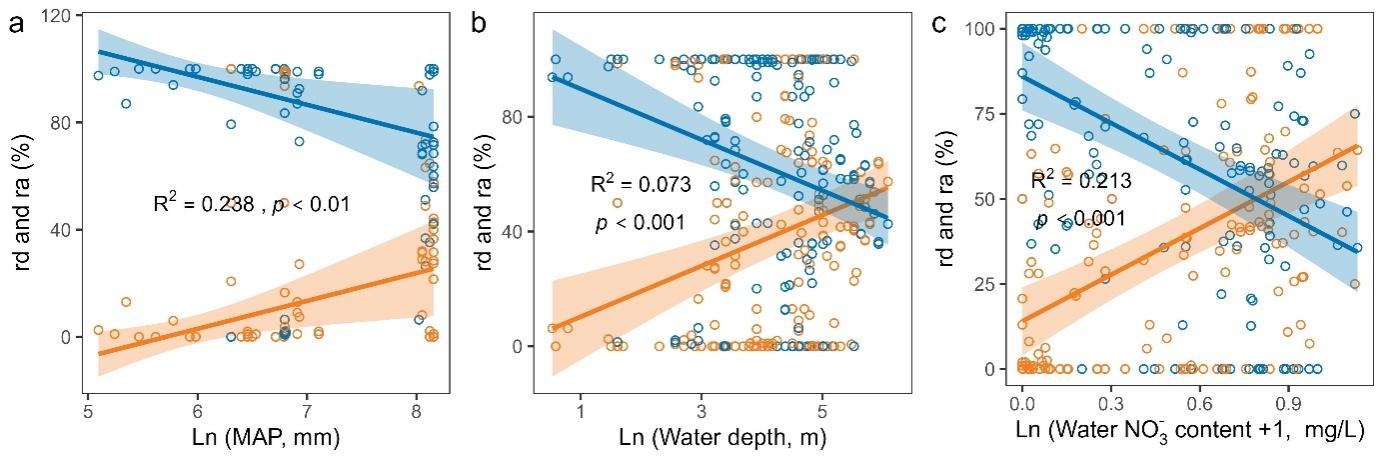


**Figure S8. Bivariate relationships between climatic factors and water parameters and the contribution of denitrification (rd, blue) and anammox (ra, yellow) in water.** The linear regression models of both rd and ra had the same statistical test results. Abbreviations: MAP, mean annual precipitation; NO_3_^-^, nitrate.


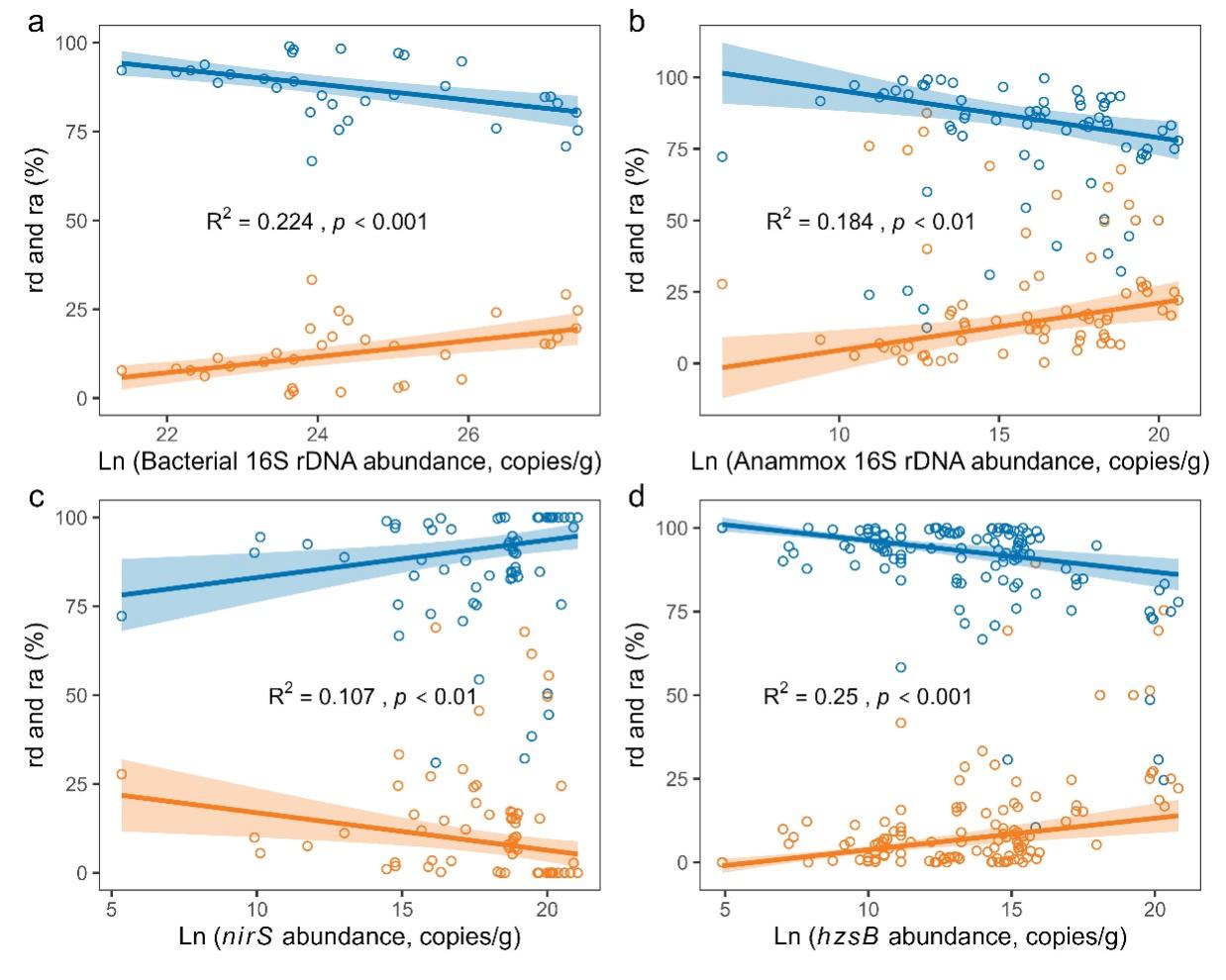


**Figure S9. Bivariate relationships between microbial gene abundance and the contribution of denitrification (rd, blue) and anammox (ra, yellow) in soils.** The linear regression models of both rd and ra had the same statistical test results. Abbreviations: *nirS*, encoding nitrite reductase; *hzsB*, encoding hydrazine synthetase.


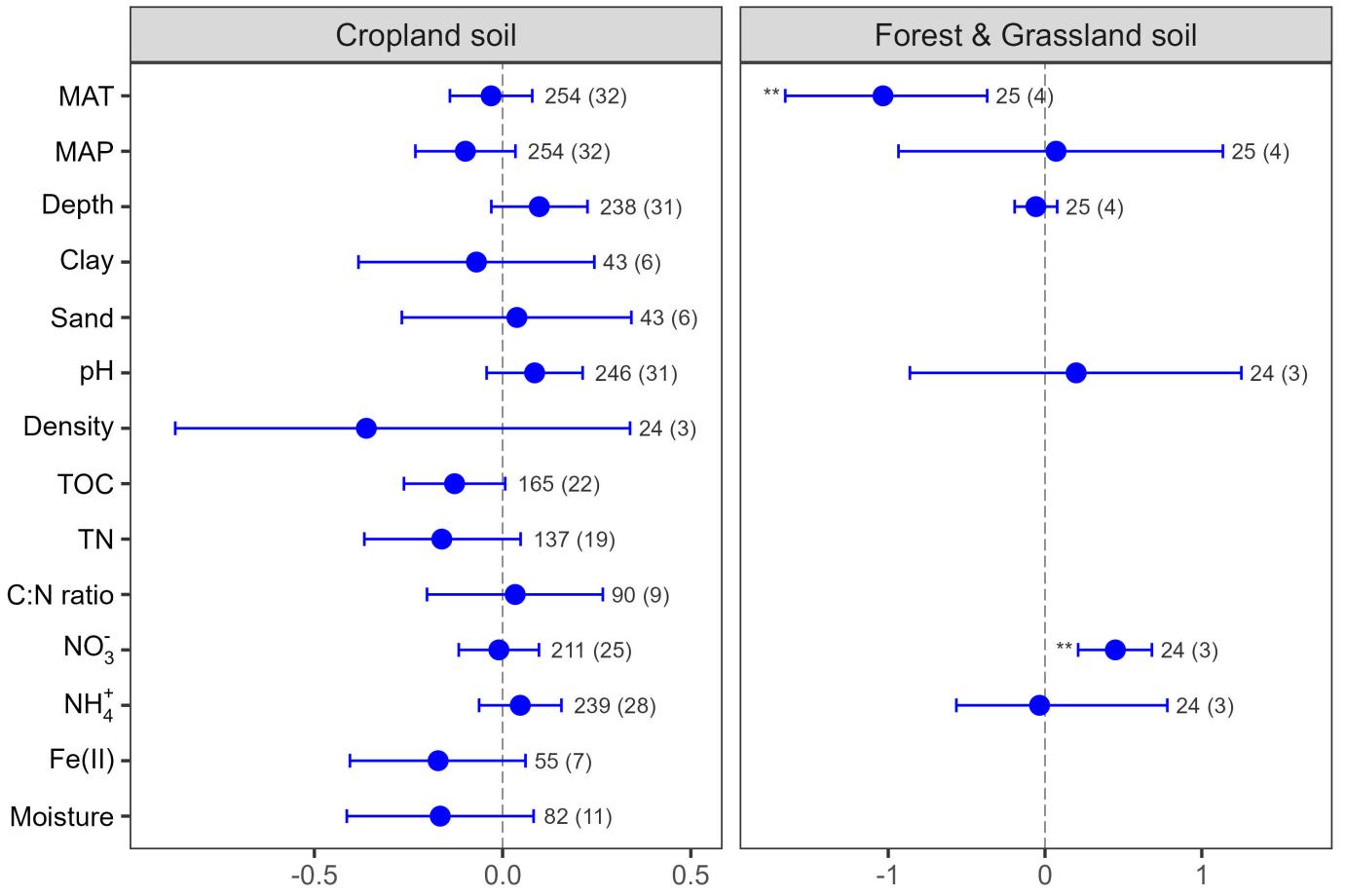


**Figure S10. The slopes of the bivariate relationships from the linear mixed effects models between the contribution of anammox to nitrogen loss and environmental factors in terrestrial soils.** The data were standardized using z-score normalization before modeling. Values are mean ± 95% confidence intervals (95% CI) of the slope between ra and each variable, and the slopes were significant when 95% CI does not include zero value (*p* < 0.05). The number of observations is outside parentheses, and the number of studies is inside parentheses. Significance: ***, *p* < 0.001; **, *p* < 0.01; *, *p* < 0.05. Abbreviations: MAT, mean annual temperature; MAP, mean annual precipitation; TOC, total organic carbon; TN, total nitrogen; C:N ratio, ratio of total carbon content and total nitrogen content; NO_3_^-^, nitrate; NH_4_^+^, ammonia; Fe(II), ferrous ion.


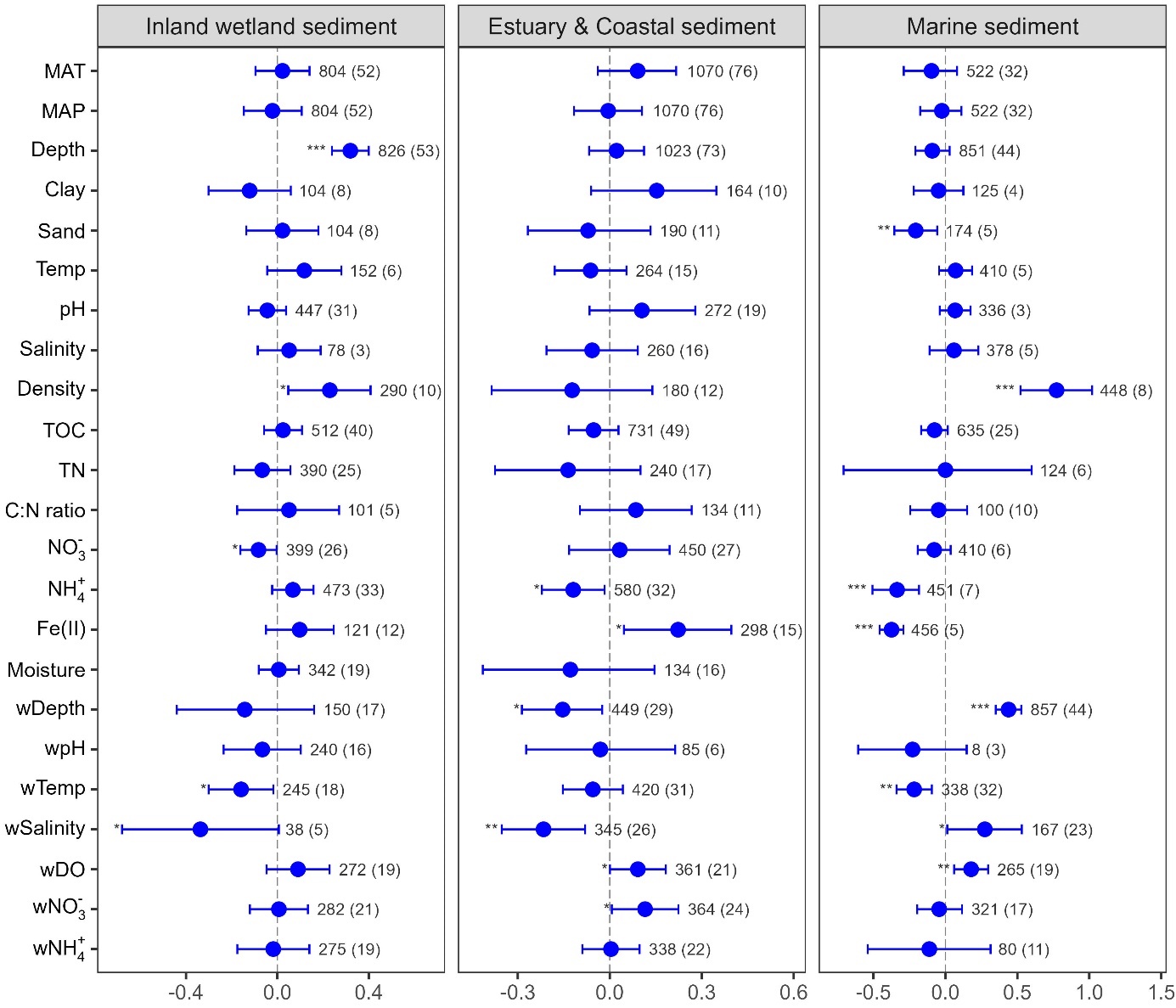


**Figure S11. The slopes of the bivariate relationships from the linear mixed effects models between the contribution of anammox to nitrogen loss and environmental factors** **in aquatic sediments.** The data were standardized using z-score normalization before modeling. Values are mean ± 95% confidence intervals (95%CI) of the slope between ra and each variable, and the slopes were significant when 95%CI does not include zero value (*p* < 0.05). The number of observations is outside parentheses, and the number of studies is inside parentheses. Significance: ***, *p* < 0.001; **, *p* < 0.01; *, *p* < 0.05. Abbreviations: MAT, mean annual temperature; MAP, mean annual precipitation; temp, temperature; TOC, total organic carbon; TN, total nitrogen; C:N ratio, ratio of total carbon content and total nitrogen content; NO_3_^-^, nitrate; NH_4_^+^, ammonia; Fe(II), ferrous ion. Variables beginning with a lowercase ‘w’ are water properties.


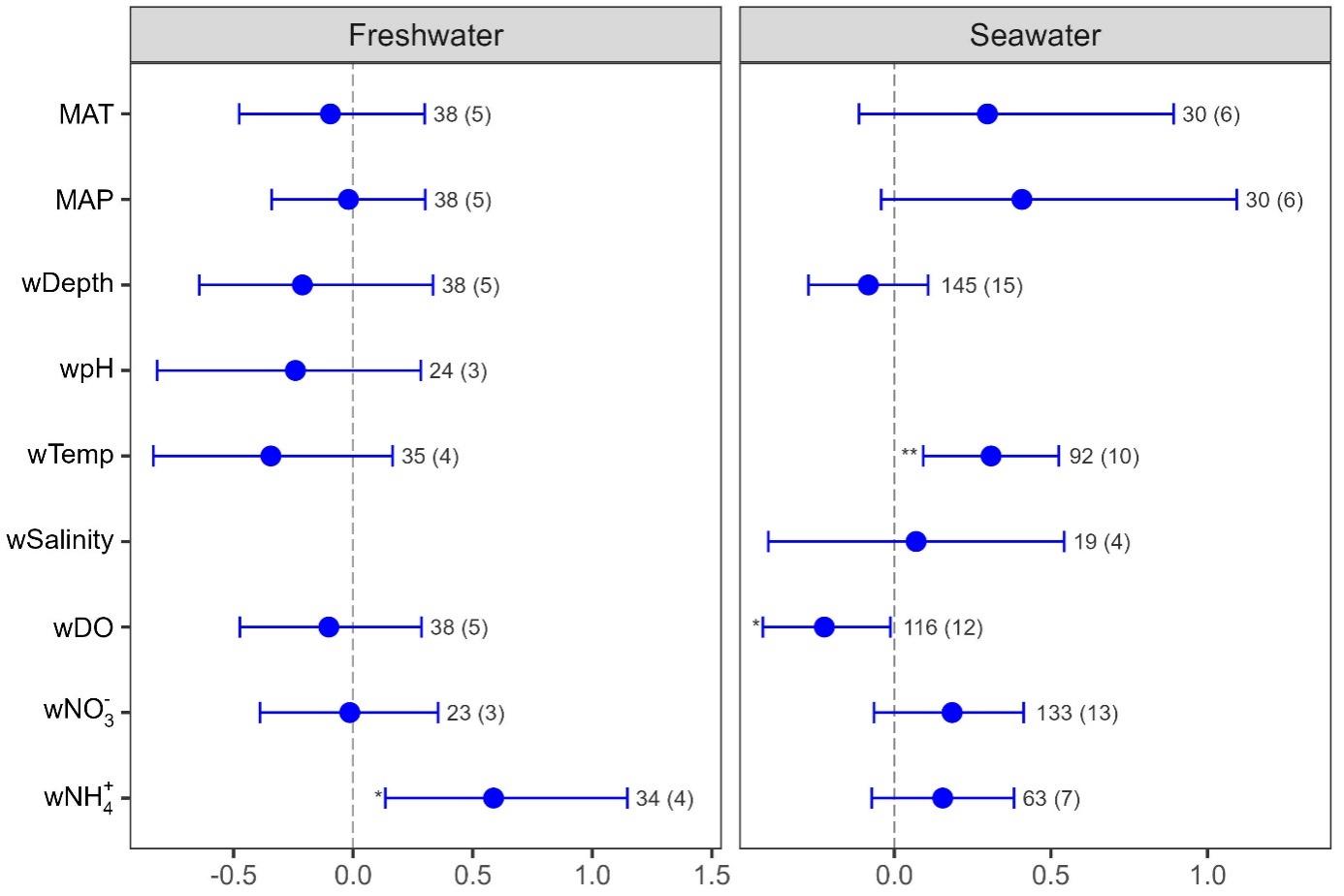


**Figure S12. The slopes of the bivariate relationships from the linear mixed effects models between the contribution of anammox to nitrogen loss and environmental factors** **in freshwater and seawater**. The data were standardized using z-score normalization before modeling. Values are mean ± 95% confidence intervals (95%CI) of the slope between ra and each variable, and the slopes were significant when 95%CI does not include zero value (*p* < 0.05). The number of observations is outside parentheses, and the number of studies is inside parentheses. Significance: ***, *p* < 0.001; **, *p* < 0.01; *, *p* < 0.05. See Figure S14 for abbreviations. The lowercase ‘w’ at the beginning represents water.


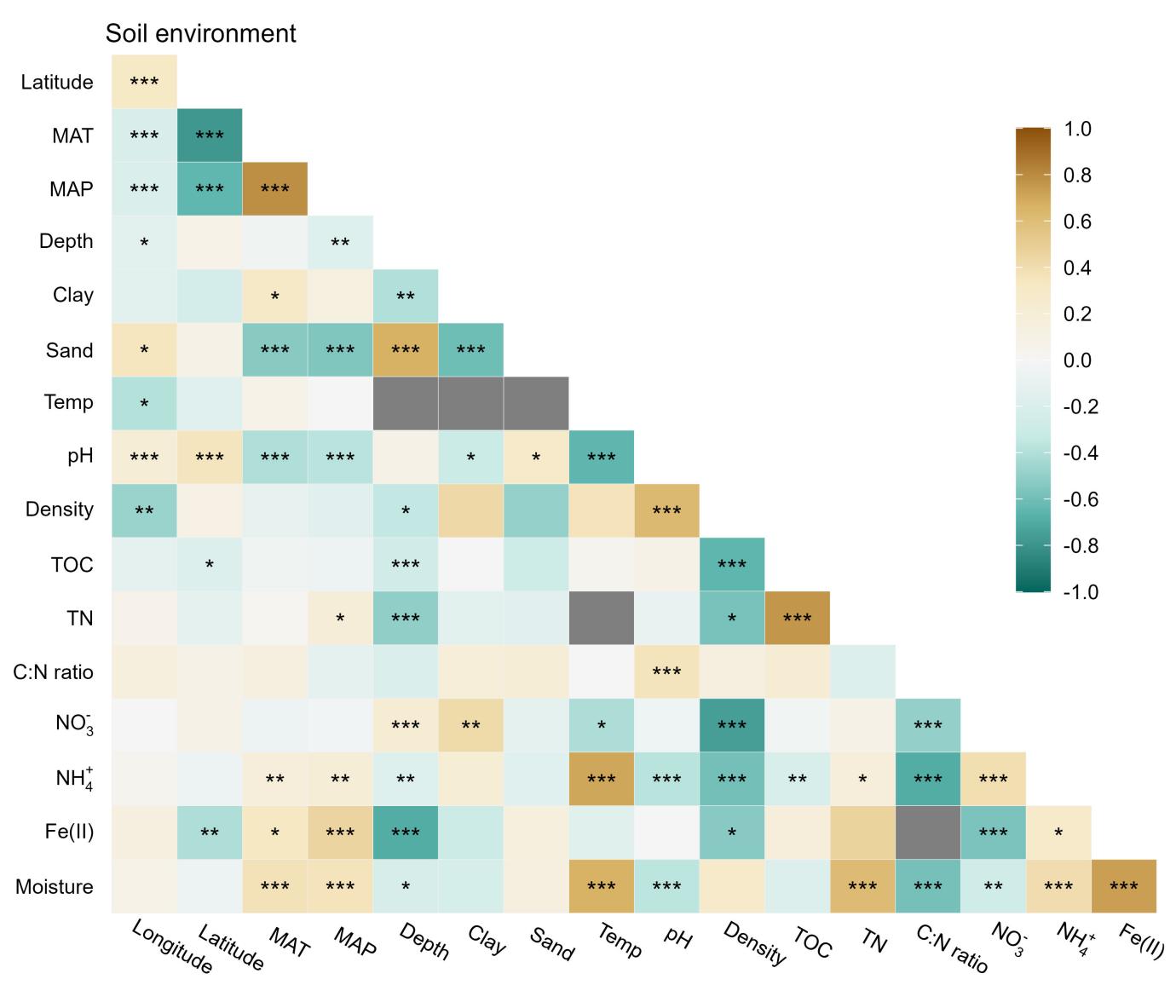


**Figure S13. Spearman correlations between geoclimatic and soil properties in soil environment.** Abbreviations: MAT, mean annual temperature; MAP, mean annual precipitation; Temp, temperature; TOC, total organic carbon; NO_3_^-^, nitrate; NH_4_^+^, ammonia; C:N ratio, ratio of total carbon content and total nitrogen content; Fe(II), ferrous ion. Significance: ***, *p* < 0.001; **, *p* < 0.01; *, *p* < 0.05.


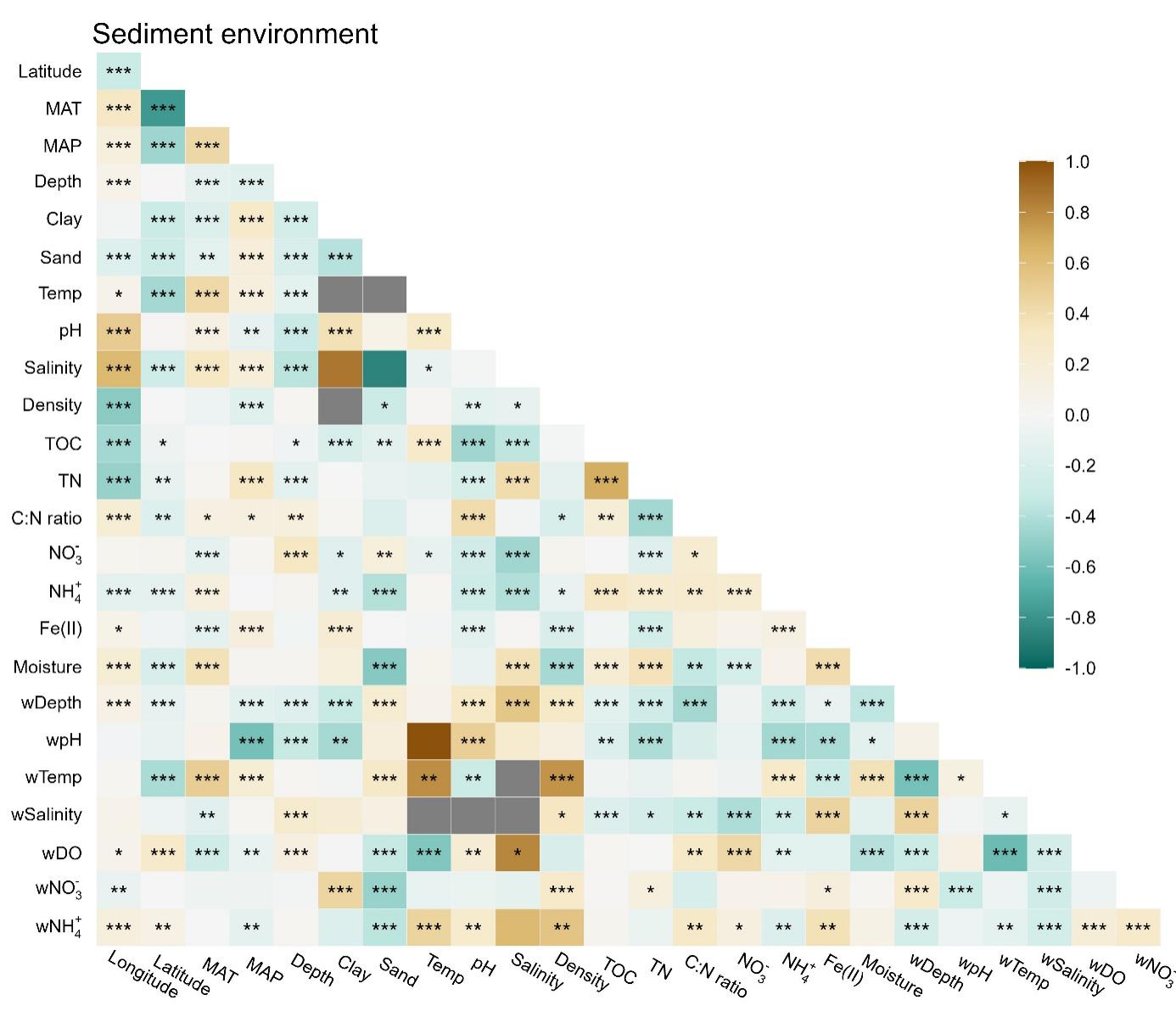


**Figure S14. Spearman correlations between geoclimatic factors, sediment and overlying water properties in sediment environments.** Abbreviations: MAT, mean annual temperature; MAP, mean annual precipitation; Temp, temperature; TOC, total organic carbon; NO_3_^-^, nitrate; NH_4_^+^, ammonia; C:N ratio, ratio of total carbon content and total nitrogen content; Fe(II), ferrous ion; DO, dissolved oxygen. Variables beginning with a lowercase ‘w’ are water properties. Significance: ***, *p* < 0.001; **, *p* < 0.01; *, *p* < 0.05.


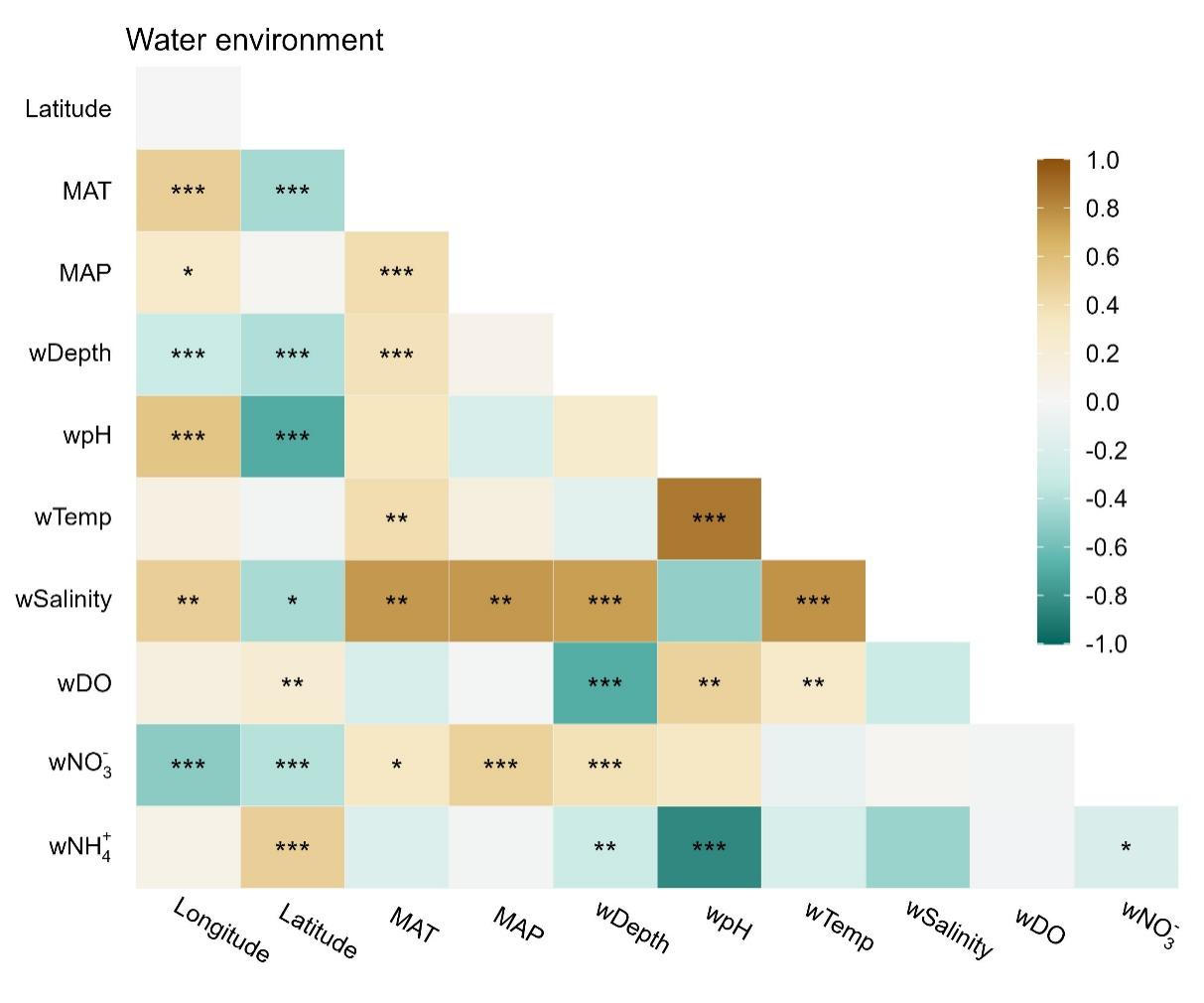


**Figure S15. Spearman correlations between geoclimatic factors and water properties in water environment.** Abbreviations: MAT, mean annual temperature; MAP, mean annual precipitation; Temp, temperature; DO, dissolved oxygen; NO_3_^-^, nitrate; NH_4_^+^, ammonia. Variables beginning with a lowercase ‘w’ are water properties. Significance: ***, *p* < 0.001; **, *p* < 0.01; *, *p* < 0.05.


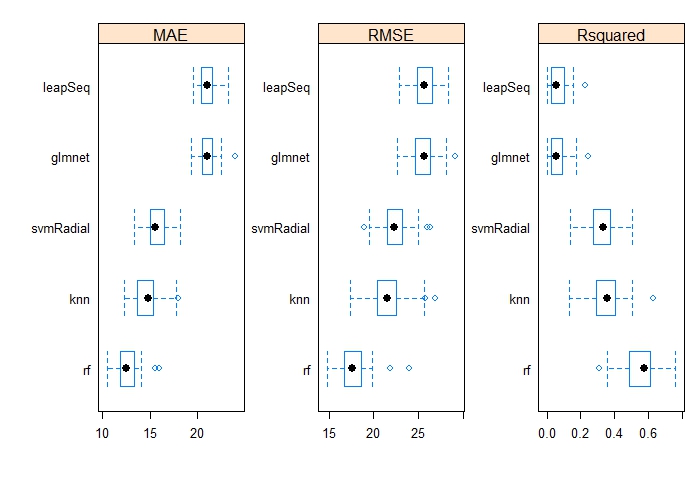


**Figure S16. Comparison of the mean of the absolute value of errors (MAE), the root mean square error (RMSE), and the regression coefficients of determination (*R*^2^) for five machine learning models**. The machine learning models included random forest (rf), support vector machine (svmRadial), k-nearest neighbor (knn), stepwise regression (leapSeq), and generalized linear models (glmnet).
